# Supplementary material for: Bile Acids Promote Hepatic Biotransformation and Excretion of Aflatoxin B1 in Broiler Chickens
Source: Toxins (Basel). 2023 Dec 9;15(12):694. doi: 10.3390/toxins15120694 (PMC10747845; doi:10.3390/toxins15120694)
Supplement: Supplementary file 1 [file toxins-15-00694-s001.zip › toxins-2737896-supplementary.pdf]

toxins

MDPI

Supplementary Materials: Bile Acids Promote Hepatic Biotransformation and Excretion of Aflatoxin B1 in Broiler Chickens

Liang Chen, Tian Wen, Aizhi Cao, Jianmin Wang, Hua Pan and Ruqian Zhao

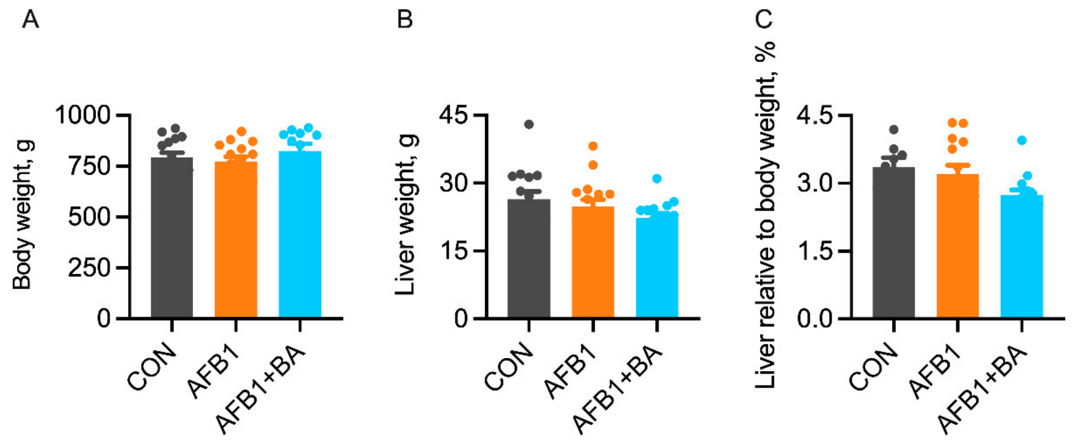

Figure S1: Effect of BA on the liver parameters in broiler chickens

A) Body weight. B) Liver weight. C) Liver relative to body weight.

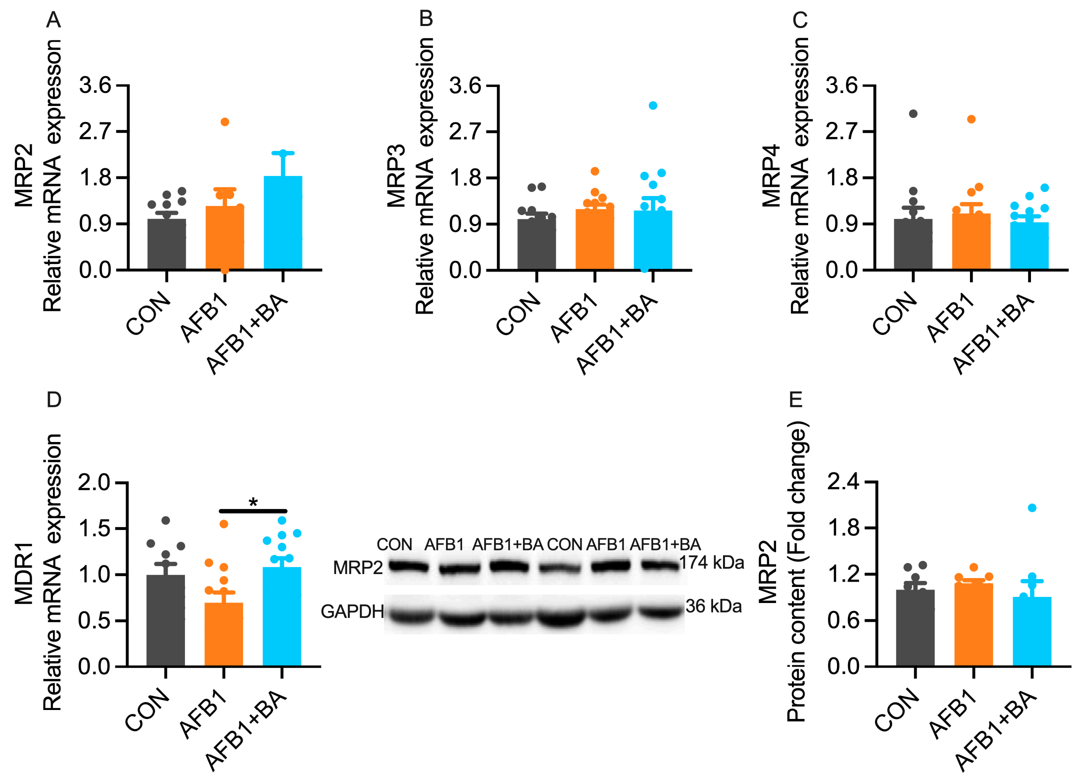

Figure S2: Effect of BA on the hepatic efflux transporters in broiler chickens

A) Hepatic mRNA expression of *MRP2*. B) Hepatic mRNA expression of *MRP3*. C) Hepatic mRNA expression of *MRP4*. D) Hepatic mRNA expression of *MRD1*. E) Hepatic protein expression of MRP2.

Table S1: Raw data for housekeeping genes

Genes name	GAPDH	ACTB	RPLPO	PPIA	18S
CON group	18.599	18.650	19.628	19.195	15.769
	19.230	16.769	17.415	18.317	15.843
	19.301	17.337	18.564	18.587	17.409
	18.168	17.495	18.909	18.849	17.630
	18.466	18.270	20.430	19.757	18.327
	19.372	15.696	17.959	17.968	16.189
	19.628	18.585	18.887	18.734	16.460
AFB1 group	19.760	18.207	21.934	18.639	16.979
	19.956	18.145	18.906	18.932	17.884
	19.447	17.956	18.883	18.962	16.730
	20.833	18.205	19.515	19.315	17.218
	19.410	18.180	19.366	18.609	18.140
	19.719	16.349	18.035	18.172	16.263
	18.810	16.161	18.193	18.371	16.200

Note: GAPDH, Glyceraldehyde 3-phosphate dehydrogenase; ACTB, Actin beta; RPLPO, Ribosomal Protein Large P0; PPIA, Peptidylprolyl Isomerase A; 18S, 18S ribosomal RNA.

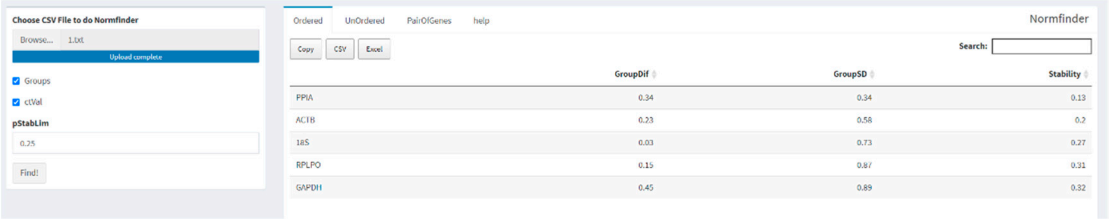

Website: https://seqyuan.shinyapps.io/seqyuan_prosper/;

Figure S3: The stability analysis of housekeeping genes
